# Calpastatin Gene (CAST) Is Not Associated with Late Onset Sporadic Parkinson’s Disease in the Han Chinese Population

**DOI:** 10.1371/journal.pone.0070935

**Published:** 2013-08-09

**Authors:** Lan Zhang, Hui Ding, Dan-Hui Wang, Yan-Li Zhang, Andrius Baskys, Piu Chan, Yu Zhong, Yan-Ning Cai

**Affiliations:** 1 Department of Pharmacology, Xuanwu Hospital of Capital Medical University, Beijing, P.R. China; 2 Department of Neurobiology, Xuanwu Hospital of Capital Medical University, Beijing, P.R. China; 3 Key Laboratory for Neurodegenerative Diseases of Ministry of Education, Beijing, P. R. China; 4 Laboratory of Chronobiology and Chronomedicine, Beijing, P. R. China; 5 Tyler Village Wellness and Recovery Clinic for Older Adults, Riverside County Department of Mental Health, Riverside, California, United States of America; 6 University of California Riverside School of Medicine, Riverside, California, United States of America; 7 Bioyong (Beijing) Technology Co., Ltd., Beijing, P. R. China; School of Medicine and Health Sciences, University of North Dakota, United States of America

## Abstract

Recent studies point to an association between the late-onset sporadic Parkinson’s disease (PD) and single nucleotide polymorphisms (SNPs) rs1559085 and rs27852 in Ca^2+^-dependent protease calpain inhibitor calpastatin (CAST) gene. This finding is of interest since loss of CAST activity could result in over activated calpain, potentially leading to Ca^2+^ dysregulation and loss of *substantia nigra* neurons in PD. We explored the association between CAST SNPs and late-onset sporadic PD in the Han Chinese population. The study included 615 evaluable patients (363 male, 252 female) with PD and 636 neurologically healthy controls (380 male, 256 female) matched for age, gender, ethnicity, and area of residence. PD cases were identified from the PD cohort of the Chinese National Consortium on Neurodegenerative Diseases (www.chinapd.cn). A total of 24 tag-SNPs were genotyped capturing 95% of the genetic variation across the CAST gene. There was no association found between any of the polymorphisms and PD in all models tested (co-dominant, dominant-effect and recessive-effect). Similarly, none of the common haplotypes was associated with a risk for PD. Our data do not support a significant association between the CAST gene polymorphisms and late onset sporadic PD in the Han Chinese population.

## Introduction

Parkinson’s disease (PD) first described by James Parkinson in 1819 as “Shaking palsy” [1] is a progressive neurodegenerative illness that affects 1.7 million people in China [2]. Estimates for idiopathic PD in the world’s ten most populated nations ranged from 4.1 to 6.6 million in 2005, with a projected expansion to 8.7 to 9.3 million by 2030 [3]. Clinical features of PD include motor symptoms such as tremor, muscular rigidity, bradykinesia, postural instability, as well as cognitive symptoms, such as dementia. Other characteristics include visual hallucinations [4] and olfactory impairment [5]. Neuropathological features of PD consist of a loss of *substantia nigra* (SN) dopaminergic neurons and widespread occurrence of Lewy bodies and dystrophic Lewy neurites [6].

Aging, genetic factors and environmental toxins are thought to contribute to the etiology of PD [7]. Approximately 5–10% of patients are now known to have monogenic forms of the disease [8] and recent genome-wide association studies (GWAS) identified several susceptibility loci albeit none of the results reached genome-wide significance [9]–[12]. Most recent studies pointed to a strong association between late-onset sporadic PD and two genetic variants (rs1559085 and rs27852) in calcium-dependent protease calpain inhibitor calpastatin (CAST) gene [13], [14]. These findings are of interest, because SN dopaminergic neurons have significant calcium-dependent pacemaker activity, and altered calcium homeostasis has been implicated PD pathogenesis [15]. In addition, over activated calpain had been implicated in destruction of cytoskeletal proteins [16] and could be potentially responsible for the loss of SN neurons in PD [17]. If confirmed in a more diverse population sample, the finding of CAST – PD association could potentially lead to development of novel PD research, treatment and prevention strategies including gene therapy [18]. We have sought to explore the association between CAST single nucleotide polymorphisms (SNPs) and late-onset sporadic PD in the Han Chinese population.

## Materials and Methods

### Subjects

PD cases used in the present study were identified from the PD cohort of the Chinese National Consortium on Neurodegenerative Diseases (CNCPD, www.chinapd.cn), established by the Chinese Parkinson Study Group (CPSG), a collaboration of 42 clinical centers managed by the coordination center at Xuanwu Hospital of Capital Medical University in Beijing. PD was diagnosed by movement disorder specialists using the United Kingdom PD Society Brain Bank Criteria [19]. Since our interest was in late-onset sporadic PD, individuals with a family history of PD in a first- or second-degree relative or individuals with the disease onset earlier than 50 years as well as those with dementia were excluded. Control subjects were selected from the community cohorts. Informed consent was obtained from all patients and controls. This study was approved by the Beijing Xuanwu hospital Ethics Committee, and was conducted in compliance with national legislation, and in accordance with the World Medical Association International Code of Medical Ethics and the Declaration of Helsinki.

### Extraction of Genomic DNA

A 2 ml volume of venous blood samples from each participant was collected. Total genomic DNA was extracted using the Whole Blood DNA Extraction Kit (Tiangen Biotech, Co., Ltd, Beijing, China), according to the manufacturer’s instructions.

### Selection of SNPs

Tag SNPs (tag-SNPs) were selected by using genotype data obtained from the International HapMap Project (http://hapmap.ncbi.nlm.nih.gov data [20] (release #27, Phase II+III Feb 09). We aimed at defining a set of tag-SNPs that had an estimated r^2^>0.8 with the untyped SNPs [21]. We used Haploview v.4.2 software (http://www.broad.mit.edu/haploview/haploview-downloads) to select the tag-SNPs that have a minor allele frequency >0.05 in CHB. For the CAST, which spans 114045 bp, 23 HapMap-based tag-SNPs were selected, including rs27852, which was reported to be associated with PD in Caucasians. The selected SNPs captured 95% of the genetic variation across the gene. We also genotyped one additional SNP (rs1559085), which was reportedly associated with PD [11]. Thus, a total of 24 SNPs were genotyped in the present study.

### Genotyping Assays

SNPs were typed using iPLEX chemistry on a matrix-assisted laser desorption/ionization time-of-flight mass spectrometer (MALDI-TOF-MS, named as MassARRAY system, Sequenom, Inc.) [21]. The procedure consisted of 3 principal steps. Step (1) was multiplex PCR amplification; PCR reactions were carried out in standard 384-well plates in 5 µl per reaction with 10 ng of genomic DNA, 0.5 units of Taq polymerase (HotStarTaq, Qiagen), 500 µmol of each deoxynucleotide triphosphate (dNTP), and 100 nmol of each PCR primer. PCR thermal cycling was carried out in an ABI-9700 instrument for 15 min at 94°C, followed by 45 cycles of 20 s at 94°C, 30 s at 56°C, and 60 s at 72°C. PCR products were electrophoresed on 2.0% agarose. Step (2) was removal of residual primers and dNTPs; after the PCR reaction, 2 µl containing 0.3 units of Shrimp Alkaline Phosphatase was added, and the reaction was incubated at 37°C for 20 min followed by inactivation for 5 min at 85°C. Step (3) was PCR with single base extension; after adjusting the concentrations of extension primers to equilibrate signal-to-noise ratios, the post-PCR primer extension reaction of the iPLEX Gold Kits (Sequenom, Inc.) assay was done in a final 9 µl volume extension reaction containing 0.2 µl (100 µmol) of termination mix, 0.04 µl containing 0.05 units of DNA polymerase (Sequenom, Inc.), and 625 to 1,250 nmol/l extension primers. A 200-short-cycle program was used for the iPLEX reaction: initial denaturation was for 30 s at 94°C followed by 5 s at 94°C and five cycles of 5 s at 52°C and 5 s at 80°C. An additional 40 annealing and extension cycles were then looped back to 5 s at 94°C, five cycles of 5 s at 52°C and 5 s at 80°C. The final extension was carried out at 72°C for 3 min and the sample was cooled to 4°C. (4) Analyses of purified extension reaction products by MALDI-TOF-MS; samples were then manually desalted by using 6 mg of clean resin and a dimple plate and subsequently transferred to a 384-well Spectro-CHIP (Sequenom, Inc.) using a nanodispenser. Mass spectrum was acquired by Compact Mass Spectrometer and analyzed by MassARRAY Typer 4.0 Software (Sequenom, Inc.). The PCR assay was arrayed with two no-template controls and four duplicated samples in each 384-well format as quality controls. All genotyping results were generated and checked by laboratory staff unaware of the patient status.

### Statistical Methods

Values are expressed as mean ± standard deviation (SD) or as numbers and percentages. Differences in age between PD group and control group were evaluated using Student’s *t*-test. Differences in frequencies of the alleles and genotypes between PD group and control group were evaluated using the *χ^2^*-test.

Hardy-Weinberg Equilibrium (HWE) was tested by the chi-square test for goodness of fit using the Technische Universitat München developed Web-based program (http://ihg.gsf.de/cgi-bin/hw/hwa1.pl) in the control group, and a P-value of <0.05 was considered to be statistically significant. We used logistic regression analysis to test for association between the CAST gene selected variants and PD risk, with adjustment for age and gender. These analyses were conducted with Stata statistical package (v. 10.0, Stata Corp LP, College Station, TX, USA). All P values are two tailed.

The pairwise linkage disequilibrium (LD) among the SNPs was examined using Lewontin’s standardized coefficient D′ and LD coefficient r^2^ [22], and haplotype blocks were defined by the method described earlier [22] using the Haploview v.4.2 software with default settings (the CI for a strong LD was minimal for upper 0.98 and low 0.7 and maximal for a strong recombination of 0.9, and a fraction of strong LD in informative comparisons was at least 0.95). PHASE 2.1 Bayesian algorithm [23] was used to estimate haplotype frequencies.

Genetic power calculator [24] was used for power calculation. Power was set at 0.8. Significance level was set at 0.05. Ratio of cases to controls was 1∶1. Allele frequency for each SNP site was set according to data generated by HapMap project [20]. PD prevalence was set at 0.02. Genotype relative risk was set according to the study of Allen et al. [14]. Accordingly, the minimal sample size for cases or control was 464 (rs1065407) or less.

## Results

### Sample Characteristics

The study included 615 evaluable patients (363 male, 252 female) with PD, and 636 neurologically healthy controls (380 male, 256 female) matched for age, gender, ethnicity, and area of residence. The patients’ mean age was 67.2±8.9 years. The mean age at PD onset was 62.1±9.2 years. The mean age of the control group was 67.5±6.5 years.

### Individual SNP Association Analysis

The SNP IDs, locations and type of the genotyped markers are given in Table 1. All genotype distributions in control group were consistent with those expected from the HWE (all P>0.05), except for rs3816555 (HWE P = 0.009). Therefore, this polymorphism was excluded from further analyses.

**Table 1:** Genotyped SNPs, their type and location.

SNP	Genome position (bp)^a^	Allele 1	Allele 2	Type of variant	P value for HWE Control PD	
rs13187079	96043366	A	G	intron	0.191	0.691
rs469633	96056500	C	G	intron	0.925	0.849
rs10053056	96069176	T	C	UTR-3	0.222	0.793
rs469532	96077746	A	C	intron	0.815	0.945
rs11739478	96079361	C	A	intron	0.301	0.141
rs26515	96080593	C	T	intron	0.173	0.162
rs27991	96083069	G	A	intron	0.140	0.230
rs151904	96092408	A	G	intron	0.416	0.420
rs13183352	96097536	A	G	intron	0.268	0.273
rs12520836	96098782	C	A	intron	0.066	0.994
rs17086611	96099817	A	G	intron	0.051	0.899
rs26506	96105158	C	T	intron	0.598	0.924
rs3816555	96109098	A	T	intron	0.009	0.778
rs731827	96112771	A	T	intron	0.809	0.489
rs26492	96114867	T	C	intron	0.751	0.817
rs27772	96115732	A	G	intron	0.450	0.662
rs27980	96123651	A	C	intron	0.640	0.945
rs27582	96123970	G	A	intron	0.540	0.806
rs27581	96133530	C	T	intron	0.532	0.151
rs28096	96135000	G	A	intron	0.528	0.424
rs1065407	96137839	A	C	intron	0.430	0.241
rs149481	96140102	T	G	intron	0.127	0.170
rs149173	96140249	A	G	intron	0.685	0.077
rs1559085	96104458	T	C	intron	1.000	0.903

a Base position according to NCBI genome build 36.3.

The allelic and genotypic distributions and P values of the 22 tag SNPs are shown in Table 2. Under the co-dominant genetic model, no SNP was significantly associated with PD. Further logistic regression analyses revealed that under the dominant-effect model, as well as recessive-effect model, no association with PD was found in any polymorphism (data not shown). Regarding the rs1559085 SNP, all of the controls were TT carriers. A total of 603 TT and 5 TC carriers were identified in the patient group, resulting in an association with marginal significance (P = 0.02), which does not survive the multiple test corrections.

**Table 2:** Allele and genotype frequencies and P values of a single-locus association in the study.

SNP	Genotype/allele	Controls N (%)	PD N (%)	P-value
rs13187079	1/1	379 (59.9)	330(55.6)	0.158
	1/2	214 (33.8)	223(37.5)	
	2/2	40 (6.3)	41 (6.9)	
	1	594 (76.5)	535(74.5)	0.309
	2	182 (23.5)	183(25.6)	
rs469633	1/1	382(60.3)	331(55.7)	0.221
	1/2	220(34.8)	226(38.0)	
	2/2	31 (4.9)	37(6.3)	
	1	601 (77.4)	540(75.2)	0.083
	2	175 (22.5)	178(24.8)	
rs10053056	1/1	114 (18.1)	123(20.6)	0.487
	1/2	325 (51.7)	292(49.0)	
	2/2	190 (30.2)	181(30.4)	
	1	553(44.0)	538(45.1)	0.558
	2	705(56.0)	654(54.9)	
rs469532	1/1	382(60.4)	333(55.0)	0.089
	1/2	220(34.8)	232(38.3)	
	2/2	30(4.7)	41(6.8)	
	1	984(77.8)	898(74.1)	0.029
	2	280(22.2)	314(25.9)	
rs11739478	1/1	41(6.5)	46(7.6)	0.652
	1/2	221(34.9)	215(35.7)	
	2/2	371(58.6)	341(56.6)	
	1	303(23.9)	307(25.5)	0.367
	2	963(76.1)	897(74.5)	
rs26515	1/1	204(32.4)	163(26.8)	0.065
	1/2	294(46.7)	320(52.6)	
	2/2	132(21.0)	125(20.6)	
	1	702(55.7)	646(53.1)	0.196
	2	558(44.3)	570(46.9)	
rs27991	1/1	88(13.6)	78(13.0)	0.092
	1/2	272(43.1)	295(49.2)	
	2/2	271(42.9)	226(37.7)	
	1	448(35.5)	451(37.6)	0.269
	2	814(64.5)	747(62.4)	
rs151904	1/1	270(42.7)	267(44.5)	0.229
	1/2	279(44.1)	273(45.5)	
	2/2	83(13.1)	60(10.0)	
	1	819(64.8)	807(67.2)	0.198
	2	445(35.2)	393(32.8)	
rs13183352	1/1	529(83.7)	495(83.2)	0.970
	1/2	96(15.2)	93(15.2)	
	2/2	7(1.2)	7(1.1)	
	1	1154(91.3)	1083(91.0)	0.801
	2	110(8.7)	107(9.0)	
rs12520836	1/1	11(1.7)	17(2.9)	0.373
	1/2	188(29.7)	167(28.1)	
	2/2	435(68.6)	411(69.1)	
	1	210(16.6)	201(16.9)	0.827
	2	1058(83.4)	989(83.1)	
rs17086611	1/1	410(64.7)	400(66.4)	0.392
	1/2	209(33.0)	182(30.2)	
	2/2	15(2.4)	20(3.3)	
	1	1029(81.2)	982(81.6)	0.794
	2	239(18.8)	222(18.4)	
rs26506	1/1	184 (28.9)	160 (26.8)	0.693
	1/2	310 (48.7)	297 (49.7)	
	2/2	142 (22.3)	140 (23.5)	
	1	678 (53.3)	617 (51.7)	0.419
	2	594 (46.7)	577 (48.3)	
rs731827	1/1	504 (79.6)	478 (78.5)	0.750
	1/2	121 (19.1)	125 (20.5)	
	2/2	8 (1.3)	6 (1.0)	
	1	1129 (89.2)	1081 (88.8)	0.734
	2	137 (10.8)	137 (11.2)	
rs26492	1/1	107 (16.8)	91 (15.2)	0.736
	1/2	303 (47.6)	288 (48.2)	
	2/2	226 (35.5)	219 (36.6)	
	1	517 (40.6)	470 (39.3)	0.495
	2	755 (59.4)	726 (60.7)	
rs27772	1/1	186 (29.3)	169 (28.8)	0.519
	1/2	324 (51.0)	287 (48.9)	
	2/2	125(19.7)	131(22.3)	
	1	696 (54.8)	625 (53.2)	0.438
	2	574(45.2)	549(46.8)	
rs27980	1/1	190 (29.9)	167 (28.3)	0.572
	1/2	320 (50.4)	293 (49.7)	
	2/2	125 (19.7)	130 (22.0)	
	1	700 (55.1)	627 (53.1)	0.325
	2	570 (44.9)	553 (46.9)	
rs27582	1/1	123 (19.3)	126 (21.3)	0.695
	1/2	322 (50.6)	291 (49.2)	
	2/2	191 (30.0)	175 (29.6)	
	1	568 (44.7)	543 (45.9)	0.548
	2	704 (55.3)	641 (54.1)	
rs27581	1/1	468 (74.2)	455 (75.6)	0.359
	1/2	153 (24.2)	132 (21.9)	
	2/2	10 (1.6)	15 (2.5)	
	1	1089 (86.3)	1042 (86.5)	0.854
	2	173 (13.7)	162 (13.5)	
rs28096	1/1	4 (0.6)	1 (0.2)	0.365
	1/2	79 (12.5)	69 (11.4)	
	2/2	551 (86.9)	534 (88.4)	
	1	87 (6.9)	71 (5.9)	0.317
	2	1181 (93.1)	1137 (94.1)	
rs1065407	1/1	544 (85.8)	524 (86.5)	0.833
	1/2	88 (13.9)	81 (13.4)	
	2/2	2 (0.3)	1 (0.2)	
	1	1176 (92.7)	1129 (93.2)	0.692
	2	92 (7.3)	83 (6.8)	
rs149481	1/1	52 (8.2)	44 (7.4)	0.751
	1/2	232 (36.6)	212 (35.5)	
	2/2	350 (55.2)	341 (57.1)	
	1	336 (26.5)	300 (25.1)	0.437
	2	932 (73.5)	894 (74.9)	
rs149173	1/1	403 (63.6)	367 (60.9)	0.094
	1/2	207 (32.6)	197 (32.7)	
	2/2	24 (3.8)	39 (6.5)	
	1	1013 (79.9)	931 (77.2)	0.103
	2	255 (20.1)	275 (22.8)	

Allele 1 and allele 2 for each maker are specified in Table 1.

### LD Analysis, Haplotype Block Structure and Haplotype Analysis

**Figure 1:**
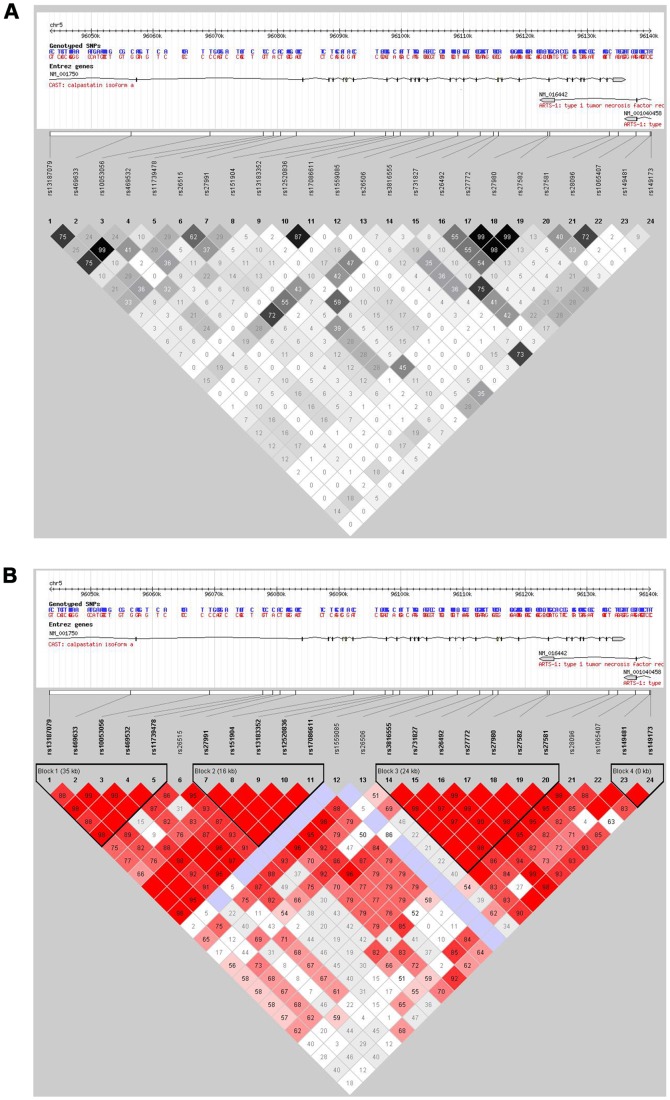
Graphical representation of the SNP locations and LD structure of the CAST gene. The SNP distribution and haplotype block structure across the CAST gene are shown, respectively. Each figure was composed of chromosome scale (the top line with even division), the transcription string (the thick bars represent exon (yellow) or UTR (blue), the thin lines represent intron), SNP scale (the hollow bar with scales representing SNP location), and graphic of LD (black-and-white, Fig. 1A) or block definition (red, Fig. 1B). The lighter and darker shades in Fig. 1A represent lower and higher values of the LD (D′) among all possible SNP pairs respectively. The numbers in squares are D′ values multiplied by 100. Haplotype blocks were defined according to the criteria by Gabriel et al. [34]. In Fig. 1B, lower values of LD (r^2^) among all possible pairs of SNPs are represented by lighter shades of color and scarlet represents higher r^2^ values. The numbers in squares are r^2^ values multiplied by 100.

The plots of the pairwise LD (D′) values for the tag-SNPs and LD structures of each gene are shown in Figure 1. We identified the following four regions of strong LD: block 1 (SNPs 1–5: size 35995 bp), covering one intronic region; block 2 (SNPs 7–11: size 16748 bp), spanning four exonic regions; block 3 (SNPs 14–20: size 22229 bp), covering 12 exonic regions and 11 intronic regions; block 4 (SNPs 23–24: size 35791 bp). Very few SNPs showed pairwise high r^2^ value, indicating that the selected tag-SNPs showed strong representation across the CAST gene. None of the common haplotypes was associated with risk for PD (data not shown).

### Discussion

The main finding of this study is a lack of a significant association between the CAST gene and late-onset sporadic Parkinson’s disease in the Han Chinese population. Haplotype analysis confirmed the lack of association in our data sample. This finding is in agreement with previously reported GWAS study results showing no association between CAST and PD [9], [10], [25], [26]. The significance of the association between the CAST polymorphism rs1559085 and PD [11] is unclear since our data indicated that this polymorphism is virtually absent in the Han Chinese population, and a low genotype frequency may obscure its statistical significance. However, it is worth noting that we did detect a marginal association in the same direction as that previously found in Caucasians. In addition, rare variants sometimes play an important role for complex disease such as PD [27], [28].

Protein encoded by CAST gene calpastatin is an endogenous calcium-dependent cysteine protease calpain inhibitor [29] with a complex mechanism of action [30]. Pharmacological reduction of calpastatin activity was shown to result in calpain over activation and dopaminergic neuron death [31]. However, unlike calpain, the calpastatin role in the regulation of dopaminergic cell death has not been unequivocally established. Low levels of calpastatin expression in the brain compared to other tissues [32] and a lack of CAST deregulation in SN neurons obtained from post-mortem PD nerve tissue by laser-capture dissection compared to controls [33] argues against the idea that calcium-dependent protease calpain inhibitor calpastatin plays a significant role in the regulation of dopaminergic cell death.

In summary, our data do not support a significant association between the CAST gene polymorphisms and late onset sporadic PD in the Han Chinese population.
